# Medial Meniscus Root Tears: Management With Single-Tunnel Repair and Meniscus Centralization

**DOI:** 10.7759/cureus.84425

**Published:** 2025-05-19

**Authors:** Ibad Shah, Ibrahim S. Majeed, Nishanth P Kurian

**Affiliations:** 1 Orthopaedic Surgery, Lifeline Multispecialty Hospital, Adoor, IND; 2 Orthopaedic Surgery, Mount Zion Medical College, Adoor, IND; 3 Orthopaedics, Lifeline Multispecialty Hospital, Adoor, IND

**Keywords:** degenerative knee, knee hip and shoulder, medial meniscus posterior root tear, meniscus tear, posterior root tear

## Abstract

Background

Meniscus root tears (MRTs) are radial tears located near the anterior or posterior meniscotibial attachment, which are often underdiagnosed and associated with accelerated knee osteoarthritis (OA). Medial meniscus posterior root tears (MMPRTs), frequently observed in middle-aged women, lead to altered knee biomechanics and joint degeneration if untreated. While historically managed with meniscectomy, the modern approach emphasizes arthroscopic repair to restore joint stability and delay OA progression.

Objective

This study evaluates the clinical outcomes of single-tunnel root repair combined with medial meniscus centralization, focusing on pain relief, functional improvement, and patient satisfaction in patients above 40 years of age.

Methods

A prospective cohort study was conducted between 2021 and 2023 at a secondary orthopedic center. Fifteen patients (mean age: 54.2 years; 86.7% female) with symptomatic MMPRTs confirmed by magnetic resonance imaging (MRI) were included. Functional outcomes were assessed using the Lysholm Knee Scoring Scale and the Western Ontario and McMaster Universities Osteoarthritis Index (WOMAC) preoperatively, and at six months and one year postoperatively. The surgical repair involved single-tunnel root fixation and medial meniscus centralization using No. 0 FiberWire. A standardized rehabilitation protocol was followed.

Results

The Lysholm score improved significantly from 45.3 ± 10.2 preoperatively to 82.3 ± 9.1 at six months and 87.6 ± 10.4 at one year. WOMAC scores decreased from 49.1 ± 10.4 to 10.7 ± 4.9 and 7.4 ± 4.1 over the same periods, reflecting reduced pain and improved function. Correlation analysis revealed no significant impact of body mass index (BMI) or varus knee alignment on outcomes, though earlier intervention (<3 months) correlated with better recovery. One patient experienced repair failure due to an incidental injury and underwent successful revision surgery.

Conclusion

Single-tunnel root repair with medial meniscus centralization is an effective surgical technique for MMPRTs in patients above 40 years, offering significant pain relief, functional improvement, and delayed OA progression. Timely diagnosis and repair are crucial for optimal outcomes, emphasizing the importance of meniscal preservation over meniscectomy.

## Introduction

Meniscus root tears (MRTs) are characterized by radial tears within 1 cm of either the anterior or posterior meniscotibial attachment or by a complete avulsion of the meniscus from its tibial attachment [1]. Historically, these injuries have been underdiagnosed and underappreciated but have gained substantial attention over recent years. The modern definition of an MRT as a “radial tear occurring within 1 cm of the root attachment of the meniscus or a complete bony or soft tissue avulsion of the root attachment altogether” was only introduced about a decade ago by Wolf Petersen and Robert Laprade [2].

Medial meniscus posterior root tears (MMPRTs) are commonly degenerative and seen in middle-aged women above the age of 50 years, representing up to 21.5% of posterior horn medial meniscus tears [3]. They are usually heralded by painful popping during light activity, such as descending stairs or walking [4]. These tears are associated with increased contact pressure and altered load distribution across the tibial plateau, leading to rapid progression of OA if untreated [5]. MRTs are often underdiagnosed in older populations, leading to persistent knee pain and accelerated joint degeneration [6,7]. This condition acts as a masquerader of early OA-inducing sequential knee joint degradation, sometimes leading to unindicated total knee replacement (TKR) if not accurately diagnosed.

Historically, MRTs were treated with total or partial meniscectomy to achieve short-term benefits [8]. Long-term follow-up studies revealed that MMPRT pullout repair was an effective intervention to protect the knee joint in terms of clinical outcomes and survivorship [9]. Therefore, the favorable treatment of MMPRT is an arthroscopic repair for cases with mild or no osteoarthritic changes. Currently, repair of meniscal root injuries is the treatment of choice to restore joint kinematics and contact pressures and delay the development of OA [10,11]. Single-tunnel pullout repair techniques are widely used due to their technical simplicity, cost-effectiveness, and adequate clinical outcomes compared to double-tunnel and anchor-based techniques [7]. Centralization of the medial meniscus has been added to reduce extrusion and enhance meniscal contact [8,12,13]. This study evaluates the outcomes of combined single-tunnel root repair and meniscus centralization, focusing on pain, function, and safety over one year.

## Materials and methods

Study design

This was a prospective cohort study conducted between 2021 and 2023 at the Lifeline Multispeciality Hospital, Adoor, India. Ethical approval was obtained from the Institutional Review Board (IRB-2021-0012), and written informed consent was obtained from all participants before inclusion.

Patient selection

A total of 15 patients aged 40 years or older with symptomatic MMPRTs confirmed by magnetic resonance imaging (MRI) were enrolled. The sample size of 15 patients was based on the availability of eligible cases meeting the strict inclusion criteria during the two-year study period at a single secondary care center. While the sample is limited, it provides initial clinical insights into the procedure's effectiveness in a specific demographic (patients aged >40 years with MMPRTs). Inclusion criteria were (1) age ≥ 40 years, (2) MRI-confirmed posterior medial MRT, and (3) ability to follow a standardized rehabilitation protocol. Exclusion criteria included (1) associated traumatic anterior cruciate ligament (ACL) injury, (2) advanced osteoarthritis (Kellgren-Lawrence Grade 3 or 4), (3) prior surgery on the same knee compartment, (4) systemic inflammatory joint disease (e.g., rheumatoid arthritis), (5) active or previous septic arthritis, and (6) neuromuscular disorders impairing knee stability or rehabilitation. High tibial osteotomy was not performed in any patient, as the degree of varus alignment was mild and did not warrant surgical correction.

Data collection

Patient demographic information, including age, sex, body mass index (BMI), knee alignment (varus angle), and osteoarthritis grade, was recorded. Functional outcomes were assessed using the Lysholm Knee Scoring Scale and the Western Ontario and McMaster Universities Osteoarthritis Index (WOMAC) preoperatively, at six months postoperatively, and one year postoperatively.

Clinical examination and MRI findings

Clinical examination for posterior meniscal root tears typically involves a thorough assessment of knee function and stability. Patients typically report pain localized to the posteromedial aspect of the knee, exacerbated by squatting or climbing stairs [9,14]. The presence of classical joint line tenderness along the posteromedial aspect of the knee which can be elicited on palpation and the restriction of terminal flexion are common findings [9,14,15].

**Figure 1:**
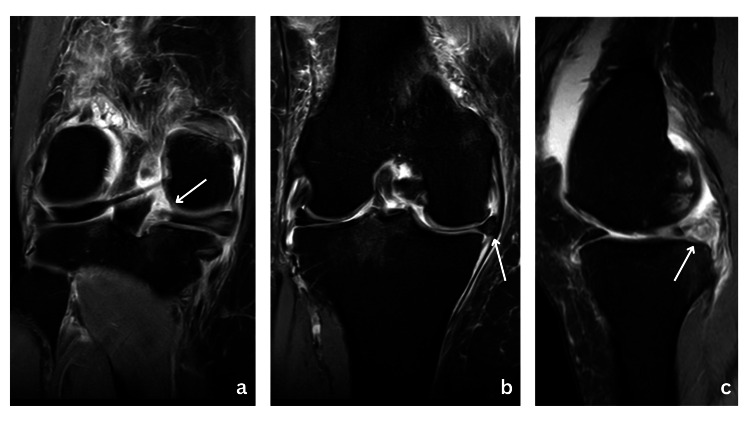
(a) Coronal MRI showing a tear at the medial meniscus root insertion (white arrow). (b) Coronal MRI highlighting extrusion of the medial meniscus, consistent with a root tear (white arrow). (c) Sagittal MRI clearly depicting a "ghost sign," nonvisualization of medial meniscus posterior root insertion (white arrow).

MRI is the gold standard for diagnosing posterior meniscal root tears [15-17]. Key MRI findings include radial tear at the posterior root, meniscal extrusion greater than 3 mm, and associated subchondral bone marrow edema or insufficiency fractures in advanced cases (Figure 1) [16-18]. A characteristic "ghost sign" on sagittal MRI sequences - where the meniscus appears faint or absent due to the radial tear - is a hallmark finding (Figure 1) [6,9,18,19]. These imaging findings are crucial not only for confirming the diagnosis but also for planning surgical repair by visualizing tear morphology and associated joint pathology.

Surgical technique

Begin by performing an examination under anesthesia, evaluating the patient's Lachman, pivot shift, drawer test, dial test, and knee stability with varus and valgus stresses. Place the patient in a supine position, and prepare and drape the injured leg in the usual sterile manner. Perform a two-portal diagnostic arthroscopy, evaluating for cartilage damage, loose bodies, and integrity of the cruciate ligaments and menisci.

If needed, to improve visualization of the medial meniscus (MM) posterior root footprint, a small resection of the medial tibial spine and a small reverse notchplasty can be performed. Using a 3.5-mm shaver and a double-sided rasp, debride the edges of the posterior root tear until bleeding occurs, in an effort to enhance healing. We debride the devascularized tissue at the edges of the tear to promote vascular infiltration. A curved arthroscopic scissor can also help release the posterior capsular attachment of the root to improve overall mobility.

**Figure 2:**
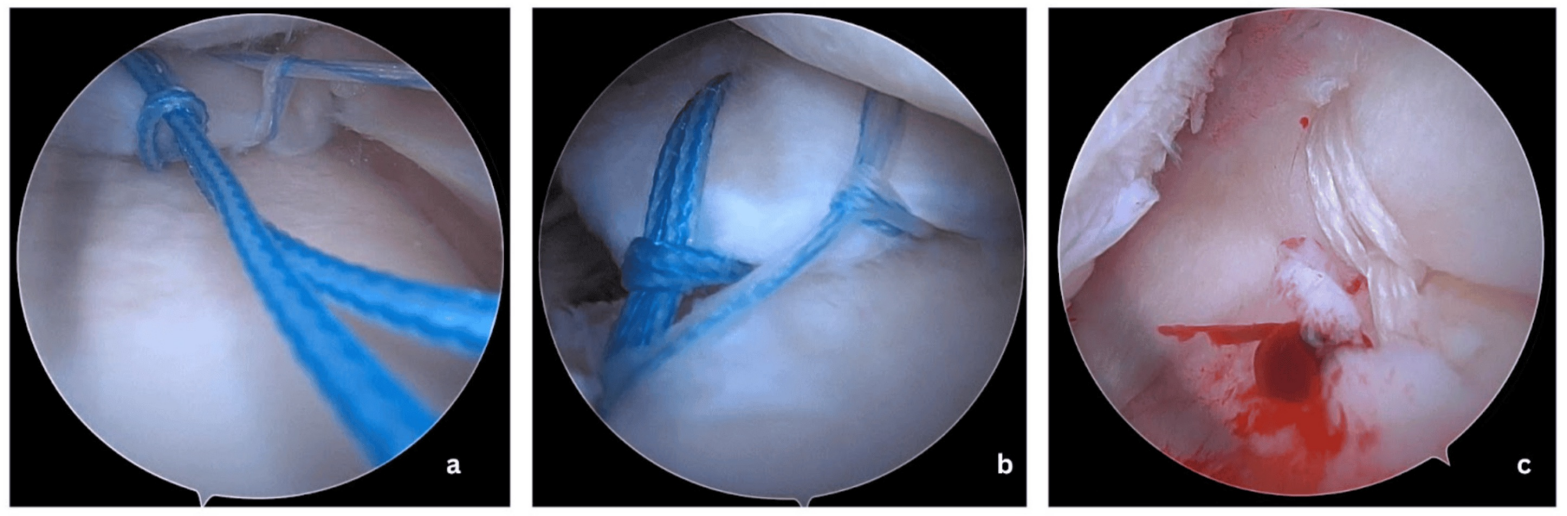
(a) Arthroscopic placement of cinch loop sutures through the torn posterior root of the medial meniscus to facilitate anatomic reattachment. (b) Sutures retrieved through a tibial tunnel, preparing for secure fixation of the posterior root to its native insertion site. (c) Placement of centralization sutures through the medial meniscus body to re-anchor and restore meniscal positioning along the tibial plateau, preventing extrusion.

Insert the Arthrex Knee Scorpion through an anteromedial portal to take two cinch loop sutures with No. 2 FiberWire through the posterior root, leaving at least a 5 mm gap between each (Figure 2, Panel a). Arthrex meniscus root repair tibial guide is then inserted through the anterolateral (AL) portal positioned behind the root attachment site. The guide pin is passed through the drill guide, and the guide is removed leaving the guide pin in position. Overdrilling using a 4.5 mm cannulated drill bit is done to widen the tunnel. The suture is then retrieved through the tunnel from the joint either using a suture retriever or suture shuttles. This will also avoid unwanted crowding of suture threads inside the joint (Figure 2, Panel b).

Now to pass the centralization suture, the viewing portal is changed to anteromedial, and the Knee Scorpion is inserted through the AL portal. One or two No. 0 FiberWire cinch loop sutures are passed through the body of the meniscus. Following this, the same root repair guide is used to create a tunnel just medial and anterior to the inner margin of the MM. The 2.7 beith pin was inserted in reverse into the tunnel, and sutures were shuttled and retrieved into the tibial end (Figure 2, Panel c).

**Figure 3:**
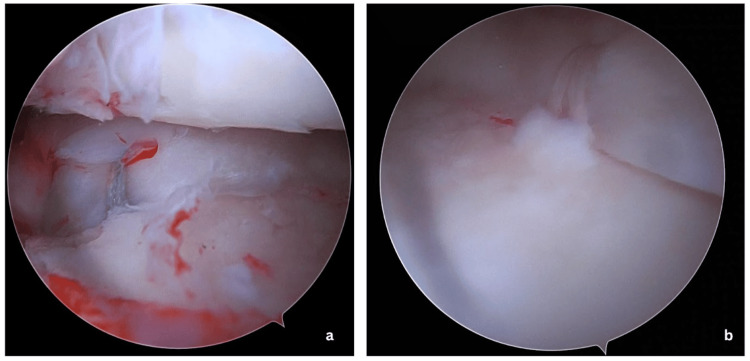
(a) Arthroscopic view showing the final construct of the single-tunnel medial meniscus posterior root repair with sutures secured through the tibial tunnel. (b) Completed medial meniscus centralization demonstrating the restoration of meniscal position and stabilization along the tibial plateau.

Initially, the root repair sutures are tightened with the knee in 70-degree flexion and tied to either an endobutton or dogbutton after adequate tensioning. Following this, the centralization stitches are tied to the root repair sutures over the bony bridge at 70-degree flexion at adequate tension. The final repair construct is shown in Figure 3.

Postoperative rehabilitation

Typically, we use a complex meniscus injury protocol for the first six weeks, where weight-bearing is completely avoided. During this time, the patient can perform quad-strengthening exercises and straight leg raises. For the first six weeks, the range of motion is limited to 90°. After six weeks, the range of motion progresses as tolerated; partial weight-bearing with an offloading brace is started, and full weight-bearing is achieved by three months. However, jogging and impact activities are delayed until six months. The patients followed a structured rehabilitation protocol. Non-weight-bearing with a restricted range of motion (limited to 90°) was advised for the first six weeks, alongside quadriceps strengthening and straight leg raises. From week 6 to 12, partial weight-bearing and progressive range of motion were permitted. Full weight-bearing was allowed after 12 weeks, while jogging and high-impact activities were delayed until at least six months postoperatively.

Data collection

Demographic and clinical variables, including age, sex, BMI, and knee alignment (measured as varus angle), were recorded. Functional outcomes were evaluated using the Lysholm Knee Scoring Scale and the Western Ontario and McMaster Universities Osteoarthritis Index (WOMAC) at baseline (preoperatively), six months, and one year postoperatively.

Statistical analysis

Descriptive statistics were used to summarize patient demographics and outcome scores. Paired t-tests were used to evaluate the changes in Lysholm and WOMAC scores between time points (preoperative versus six months, and preoperative versus one year). For each comparison, the test statistic (t-value) and corresponding p-value were reported to indicate statistical significance.

Multivariate analysis was not feasible due to the small sample size. Potential confounders such as BMI, varus alignment, and duration from symptom onset to surgery were assessed using correlation analysis, with a significance threshold set at p < 0.05. Formal power analysis and MCID (minimal clinically important difference) assessment were not performed, given the exploratory nature of the study, the limited sample size, and variability in baseline stratification and scoring sensitivity among the older patient population. Also, cartilage status was not graded in this study to maintain focus on the primary objective of evaluating functional outcomes post MM root repair with centralization.

## Results

Patient demographics

A total of 15 patients were included in the study, with a mean age of 54.2 years (range: 46-64 years). The majority were female (86.7%), and the mean BMI was 25.3 kg/m². Most patients had mild-to-moderate osteoarthritis, with 40% showing Kellgren-Lawrence Grade 2 changes and 33.3% showing Grade 1 changes (Table 1).

**Table 1:** Patient demographics and osteoarthritis grade distribution

Characteristics	Values
Age (years)	Mean: 54.2 (Range: 46-64)
Sex distribution	86.7% Female, 13.3% Male
BMI (kg/m²)	Mean: 25.3 (Range: 20.7-27.7)
Osteoarthritis grade	40% Grade 2, 33.3% Grade 1, 26.7% Normal

Functional outcomes

Patients demonstrated significant improvement in both Lysholm and WOMAC scores over time. The Lysholm score increased steadily at both six months and one year, indicating functional recovery. Similarly, the WOMAC score showed a substantial reduction, reflecting improved pain and joint function. All comparisons were statistically significant (Table 2).

**Table 2:** Statistical analysis of Lysholm and WOMAC scores over time WOMAC: Western Ontario and McMaster Universities Osteoarthritis Index.

Comparison	Lysholm score (Mean ± SD)	t-value	p-value	WOMAC score (Mean ± SD)	t-value	p-value
Pre-op vs 6 months	45.3 → 82.3 ± 9.1	-12.62	4.89 × 10⁻⁹	49.1 → 10.7 ± 4.9	20.45	7.96 × 10⁻¹²
Pre-op vs 1 year	45.3 → 87.6 ± 10.4	-14.46	8.24 × 10⁻¹⁰	49.1 → 7.4 ± 4.1	20.02	1.05 × 10⁻¹¹
6 months vs 1 year	82.3 → 87.6	-7.72	2.05 × 10⁻⁶	10.7 → 7.4	3.37	0.0047

To further illustrate the clinical improvement following single-tunnel MM root repair with centralization, line graphs were generated for both Lysholm and WOMAC scores at three key time points: preoperative, six months postoperative, and one year postoperative.

**Figure 4:**
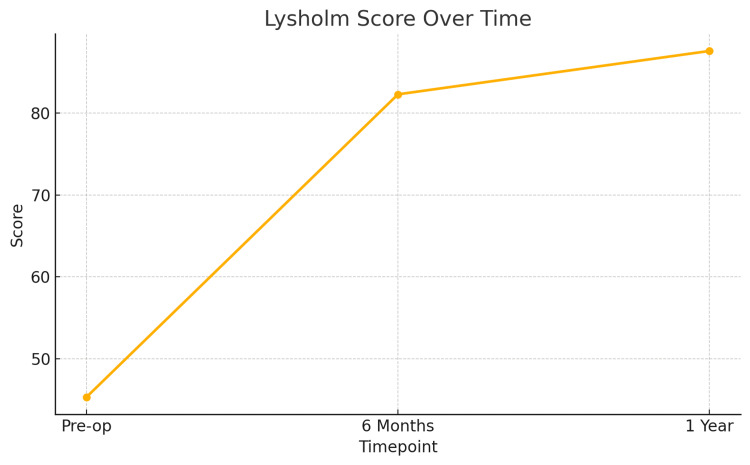
Line chart of Lysholm Knee Scores over time Line chart showing progressive improvement in the Lysholm Knee Scores at baseline (preoperative), six months postoperative, and one year postoperative. The mean score increased from 45.3 at baseline to 82.3 at six months and 87.6 at one year, indicating significant functional recovery.

**Figure 5:**
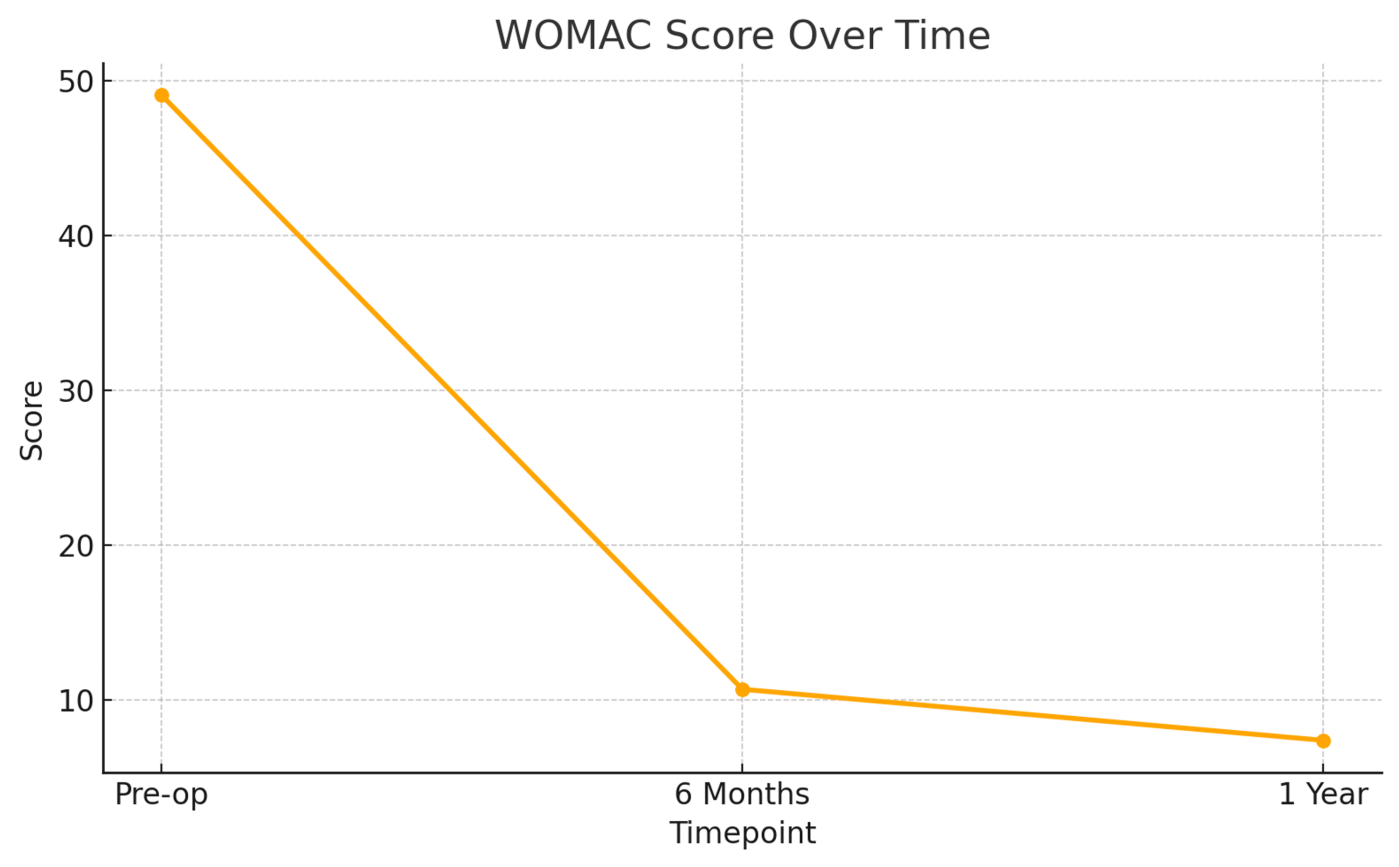
Line chart of WOMAC scores over time Line chart showing declining WOMAC scores across the same time intervals. The mean score reduced from 49.1 preoperatively to 10.7 at six months and 7.4 at one year, suggesting a substantial reduction in knee pain and improvement in joint function. WOMAC: Western Ontario and McMaster Universities Osteoarthritis Index.

Figure 4 demonstrates the progression in Lysholm Knee Scores, showing a consistent upward trend from a baseline mean of 45.3 to 82.3 at six months and 87.6 at one year, reflecting enhanced knee stability and function. Figure 5 presents the WOMAC scores, which declined from a preoperative mean of 49.1 to 10.7 at six months and further to 7.4 at one year, indicating a significant reduction in pain and improvement in joint-related quality of life.

Correlation analysis

There was no significant correlation between BMI and preoperative Lysholm or WOMAC scores, indicating that BMI did not influence baseline functional status. Notably, 86.7% of patients had a BMI ≥ 25.

Varus knee alignment (≤87°), observed in 80% of patients, did not significantly affect postoperative outcomes (p > 0.05). Patients undergoing surgery within three months of symptom onset demonstrated better improvements than those with delayed intervention, underscoring the value of early surgical management.

Complications

One patient (6.7%) experienced repair failure due to a fall three months after surgery. The patient underwent revision root repair and achieved functional recovery comparable to the rest of the cohort at the final follow-up.

## Discussion

This study demonstrates that single-tunnel root repair combined with MM centralization using No. 0 FiberWire significantly improves knee function, reduces pain, and enhances quality of life in patients above 40 years of age. The marked improvements in both Lysholm and WOMAC scores at one-year follow-up highlight the procedure’s effectiveness in restoring knee stability and delaying OA progression.

Previous studies have established that meniscal root repair enhances joint biomechanics and helps slow the progression of osteoarthritis. More recent evidence suggests that augmenting MM root repair with centralization can further reduce meniscal extrusion and improve tibiofemoral contact mechanics when compared to root repair alone, although definitive long-term clinical advantages are yet to be confirmed [12,13]. Chung et al. reported a 32-point improvement in Lysholm scores following root repair, compared to 12 points with partial meniscectomy, emphasizing the benefits of meniscal preservation [20]. Our findings of a 42.3-point improvement reinforce these results, particularly in older adults for whom joint preservation is crucial.

Although Feucht et al. noted that complete healing is seen in only 62% of patients on postoperative MRI or arthroscopy [21], our study observed consistent clinical improvement despite the absence of imaging, suggesting that substantial functional recovery is possible even without confirmed biological healing.

Varus alignment, a known risk factor for MMPRTs, was present in 80% of our cohort. However, it did not significantly affect postoperative outcomes, which is in line with LaPrade et al.'s conclusion that varus malalignment increases tear susceptibility but is not a contraindication for repair [22].

Similarly, while 86.7% of patients had a BMI ≥ 25, no significant association was found between BMI and either baseline function or postoperative recovery. This supports Nie et al.’s findings that higher BMI may influence radiologic healing, but functional improvement is achievable with appropriate rehabilitation [23]. These results suggest that patients with elevated BMI can benefit from repair and should not be excluded based solely on weight.

Our cohort also showed notable improvements among patients with early OA (grades 1 and 2), supporting the value of repair even in the presence of mild degeneration. Hurmuz et al. reported better outcomes and lower OA progression with repair versus meniscectomy, reinforcing the principle of preserving the meniscus in early OA [24].

Timely surgical intervention emerged as a key factor. Patients operated on within three months of symptom onset experienced greater improvements, which is consistent with Furumatsu et al.’s observation that early repair leads to superior results [25].

Taken together, our findings support single-tunnel root repair with centralization as an effective, broadly applicable technique regardless of BMI, varus alignment, or early degenerative changes. Early diagnosis and intervention are crucial, especially in high-risk populations, to optimize outcomes and delay OA progression.

This study is limited by small sample size, lack of imaging to confirm meniscal healing, and a predominantly female cohort, which may limit generalizability. The lack of a control group in this study limits the ability to draw causal inferences. Future randomized controlled trials are recommended to validate these findings. Additionally, the one-year follow-up restricts conclusions about long-term outcomes and the potential need for future surgical interventions.

## Conclusions

Single-tunnel root repair combined with MM centralization using No. 0 FiberWire is an effective surgical technique for treating posterior meniscal root tears in patients above 40 years of age. The significant improvements in functional scores and reduction in pain at one-year follow-up demonstrate the potential of this approach to restore knee stability, reduce symptoms, and delay the progression of osteoarthritis. While our findings suggest favorable outcomes, the small sample size and lack of imaging confirmation warrant cautious interpretation. Larger, controlled studies are needed to confirm these preliminary results.
